# Birth weight, leptin and adiponectin in patients initiating clozapine

**DOI:** 10.1192/j.eurpsy.2024.299

**Published:** 2024-08-27

**Authors:** L. Ilzarbe, M. Garriga, C. Oliveira, M. Gómez-Ramiro, A. Mallorquí, V. Ruiz-Cortés, Y. Rivas, S. Amoretti, G. Mezquida, D. Ilzarbe, E. Vieta, E. Parellada, I. Baeza, C. García-Rizo

**Affiliations:** ^1^Bipolar and Depressive Disorders Unit, Hospital Clinic, Institute of Neuroscience, University of Barcelona; ^2^Institut d’investigacions Biomèdiques August Pi i Sunyer (IDIBAPS); ^3^Centro de Investigación Biomédica en Red de Salud Mental (CIBERSAM), Instituto de Salud Carlos III, Barcelona, Spain; ^4^University of Coimbra, Coimbra, Portugal; ^5^Hospital Alvaro Cunqueiro, SERGAS, Translational Neuroscience Research Group, Galicia Sur Health Research Institute (IISGS), Vigo; ^6^Department of Psychiatry, Institute of Neuroscience, Hospital Clinic Barcelona, University of Barcelona; ^7^Barcelona Clinic Schizophrenia Unit, Hospital Clinic of Barcelona, Hospital Clinic, Institute of Neuroscience, University of Barcelona; ^8^Group of Psychiatry, Mental Health and Addictions, Vall d’Hebron Research Institute (VHIR), Psychiatric Genetics Unit, Vall d’Hebron Research Institute (VHIR); ^9^Department of Basic Clinical Practice, Pharmacology Unit, University of Barcelona; ^10^Child and Adolescent Psychiatry and Psychology Department, 2021SGR01319, Institute Clinic of Neurosciences, Hospital Clínic of Barcelona, Barcelona, Spain

## Abstract

**Introduction:**

Psychotic patients often require pharmacological treatment, which may prove ineffective, leading to treatment-resistant psychosis necessitating the use of clozapine. However, the emergence of side effects can result in discontinuation, potentially triggering a relapse of psychotic symptoms. One significant side effect is antipsychotic-induced weight gain which, over time, can lead to adverse metabolic events. Recent translational research is evaluating the impact of prenatal factors on the metabolic outcomes of psychotic patients, using a surrogate marker of the intrauterine milieu such as birth weight (BW).

**Objectives:**

We aim to evaluate the changes in leptin, adiponectin, and insulin levels in patients with treatment-resistant psychosis who initiate clozapine treatment due to persistent psychotic symptoms.

**Methods:**

Subjects older than 18 years with a diagnostic of a major mental disorder and initiating clozapine were enrolled in this 18-months longitudinal study. Neurohormones levels, including leptin, adiponeptin, and insulin were measured at baseline, 8 and 18 months during follow-up. Statistical analysis were conducted by using a fixed-effects model.

**Results:**

A total of 23 subjects initiating clozapine were evaluated during the initial mandatory 18-week period. Neurohormones, specifically leptin and adiponectin, were measured at three time points: baseline, 8 weeks, and 18 weeks. The changes in leptin levels were significantly associated with birth BW with sex differences, being inversely correlated only in females. Adiponectin was significantly associated with BW, being inversely correlated in males. Conversely, there was no observed association between insulin levels and BW.

**Conclusions:**

Our findings highlight the significance of prenatal factors in influencing the subsequent evolution of neurohormones in individuals initiating clozapine treatment. This suggests that subjects with lower BW tend to exhibit elevated neurohormone values, emphasizing the role of prenatal events in this context.

**Disclosure of Interest:**

None Declared

